# Emergency Tracheal Intubation in Patients with COVID-19: A Single-center, Retrospective Cohort Study

**DOI:** 10.5811/westjem.2020.2.49665

**Published:** 2021-05-17

**Authors:** Andrew Hawkins, Stephanie Stapleton, Gerardo Rodriguez, R. Mauricio Gonzalez, William E. Baker

**Affiliations:** * Boston University Medical Center, Department of Emergency Medicine, Boston, Massachusetts; † Boston University, Department of Anesthesiology, Boston, Massachusetts

## Abstract

**Introduction:**

The objective of this study was to compare airway management technique, performance, and peri-intubation complications during the novel coronavirus pandemic (COVID-19) using a single-center cohort of patients requiring emergent intubation.

**Methods:**

We retrospectively collected data on non-operating room (OR) intubations from February 1–April 23, 2020. All patients undergoing emergency intubation outside the OR were eligible for inclusion. Data were entered using an airway procedure note integrated within the electronic health record. Variables included level of training and specialty of the laryngoscopist, the patient’s indication for intubation, methods of intubation, induction and paralytic agents, grade of view, use of video laryngoscopy, number of attempts, and adverse events. We performed a descriptive analysis comparing intubations with an available positive COVID-19 test result with cases that had either a negative or unavailable test result.

**Results:**

We obtained 406 independent procedure notes filed between February 1–April 23, 2020, and of these, 123 cases had a positive COVID-19 test result. Residents performed fewer tracheal intubations in COVID-19 cases when compared to nurse anesthetists (26.0% vs 37.4%). Video laryngoscopy was used significantly more in COVID-19 cases (91.1% vs 56.8%). No difference in first-pass success was observed between COVID-19 positive cases and controls (89.4% vs. 89.0%, p = 1.0). An increased rate of oxygen desaturation was observed in COVID-19 cases (20.3% vs. 9.9%) while there was no difference in the rate of other recorded complications and first-pass success.

**Discussion:**

An average twofold increase in the rate of tracheal intubation was observed after March 24, 2020, corresponding with an influx of COVID-19 positive cases. We observed adherence to society guidelines regarding performance of tracheal intubation by an expert laryngoscopist and the use of video laryngoscopy.

## INTRODUCTION

A critical complication of coronavirus 2019 (COVID-19) is acute respiratory insufficiency requiring supplemental oxygen and mechanical ventilation. A recent report from Wuhan, China, found that 14% of patients with novel coronavirus infections developed acute hypoxemic respiratory failure and 2.3% of patients required endotracheal intubation (ETI).^1^ During the previous severe acute respiratory syndrome coronavirus-1 (SARS-CoV-1) epidemic in 2003, a group in Singapore analyzed airway registry data and noted a decrease in the proportion of intubations performed by trainees and anesthesiologists compared to emergency physicians (EP).^2^ A recent observational study of emergent tracheal intubation in patients with COVID-19 in two hospitals in Wuhan reported a first-pass success (FPS) rate of 89% in patients who were intubated using rapid sequence induction (RSI) and found that hypoxia and hypotension were common peri-intubation complications.^3^ To our knowledge, there has not been an analysis in the United States of airway registry data with attention to outcomes including FPS rate and complications, guideline adherence, or change in practice patterns due to resource scarcity.

Endotracheal intubation (ETI) in suspected COVID-19 cases presents challenges related to rapid patient decline, infection control, and resource scarcity. Several groups in China and Italy have described precipitous decline in oxygen saturation after loss of spontaneous breathing, particularly in patients with decreased respiratory reserve.^4^–^6^ Non-elective intubations performed in the emergency department (ED) and intensive care unit (ICU) settings have been associated with increased incidence of complications.^7^ During airway management in suspected COVID-19 patients, enhanced personal protective equipment (PPE) is ideally provided to all operators, but the use of PPE has been associated with decreased intubation success rate in simulation studies.^4^,^8^–^10^

Recent expert consensus guidelines from the Safe Airway Society recommend that the airway operator be the most experienced clinician available and, in anticipated difficult cases, the intervention should be performed by an anesthesiologist.^11^ In the 2003 SARS epidemic, an increased proportion of intubations were performed by anesthesiologists compared to EPs and fewer interventions were performed by trainees.^2^ The authors did not note a difference in intubation success or complication rates. The COVID-19 epidemic, however, is greater in scale and has led to considerable strain on healthcare systems.^6^,^12^,^13^ It is feasible that the incidence of multiple airway attempts and complications may be impacted by the current crisis, although this has not been directly studied.

## METHODS

### Outcomes

First-pass success is an important measure of patient prognosis following ETI.^14^ We have collected all emergent intubations performed at our institution as a part of an ongoing quality and patient safety initiative. The goal of this study was to understand the effect of the increased number of COVID-19 cases and its effect on FPS and risk of adverse events at our institution. We hypothesized that the complexity, volume, and environmental constraints during the COVID-19 epidemic would result in reduced FPS and increased complication rates. The secondary goals of this study were to report on the proportion of intubations performed by specialty and training level, and adherence to recently proposed guidelines for airway management in COVID-19 patients.^5^,^8^,^11^

### Study Design

This is an unmatched retrospective cohort analysis of all ETIs prospectively recorded in a continuous quality improvement database from February 1–April 23, 2020. All patients who underwent out-of-operating room (OR) ETI were included, as the note is specifically used for emergent procedures. This project was granted exemption by our institutional review board as it is an analysis of a quality improvement database. All patient name and health record numbers were made anonymous to the researchers.

**Population Health Research Capsule**

What do we already know about this issue?

*Coronavirus disease (COVID-19) prompted changes to intubation processes, primarily aimed at infection prevention. First-pass success rate is an accepted intubation performance indicator*.

What was the research question?

*We sought to compare first-pass success intubation rates prior to the COVID-19 pandemic surge to those of COVID-positive cases during the surge*.

What was the major finding of the study?

*There was a significant increase in intubations in response to COVID-19 with no difference in first-pass success rate*.

How does this improve population health?

*COVID patient intubations can be performed effectively as measured by first-pass success, even with additional infection control and process changes*.

### Study Setting and Population

We conducted this study in a 514-bed academic medical center and safety-net hospital in Boston, Massachusetts. The majority of intubations are typically performed by residents in both the emergency medicine (EM) and anesthesiology residency programs using either a direct or video laryngoscope. All intubations performed in the ED and ICU are supervised by attending physicians. The department of anesthesiology transitioned the airway response team to be comprised of expert laryngoscopists including nurse anesthetists (CRNA), anesthesiologists, and select senior residents. Prior to February 1, 2020, CRNAs did not regularly perform emergent intubations. No changes were made to the constitution of the airway response team in the ED.

### Airway Management Policy

Multidisciplinary meetings were held on best practice in airway management of COVID-19 patients at our institution in late February 2020. A consensus was reached to include routine use of rocuronium with propofol for longer paralysis to minimize disconnection of the breathing circuit and video laryngoscopy to maximize FPS in suspected COVID-19 patients (Appendix A). All patients requiring emergent intubation after February 1 were treated as possible COVID-19 patients, given the extended turnaround period for testing early in the pandemic. This involved standard use of negative pressure rooms, PPE involving an N95 mask or approved respirators, face shield, gown, and gloves. Recommendations for laryngoscopy technique and pharmacologic agents for RSI was communicated in an email and lecture format to all faculty and residents.

### Study Protocol

**Figure 1:**
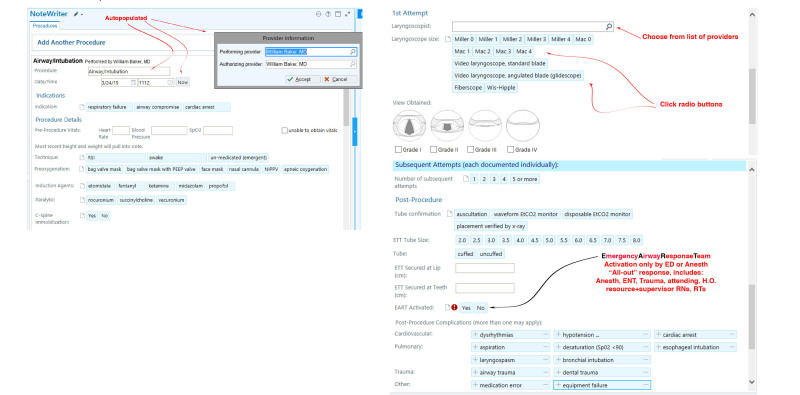
Screenshots of electronic health record airway procedure note that is created for each endotracheal intubation. The patient name, time and author are auto-populated fields. The clinician is prompted to document each attempt individually and select multiple post-procedure complications.

An airway procedure note user interface and reporting system was designed with structured and free-text fields integrated within our hospital’s electronic health record, Epic (Epic Systems Corporation, Verona, WI). Specific variables are auto-populated including patient identifier, date-time, author, and specialty. Provider type is collected as anesthesiologist, attending EP, CRNA, or resident. The primary airway operator then must document the following: technique; pre-oxygenation; induction and paralytic agent; C-spine immobilization; laryngoscopist; laryngoscope size; grade of view obtained; external laryngeal manipulation; and common post-procedure complications (dysrhythmias, hypotension, cardiac arrest, aspiration, desaturation, esophageal intubation, laryngospasm, bronchial intubation, airway trauma, dental trauma, medication error, equipment failure) (Figure 1).

The adverse events in the procedure note coincide with those identified in the NEAR study and were defined according to the intubating clinician’s discretion.^15^ Each intubation attempt is documented individually. The notes included in this study were subject to routine professional billing procedures and manual chart review by an external contractor, which prompts staff to complete documentation. For notes filed between February 1–April 23, 2020, data on EPIC intubation reports was run, which includes both internal and external lab results corresponding to COVID-19 status. The collected data was anonymized and exported into a database compliant with the Health Insurance Portability and Accountability Act of 1996. Duplicate entries were removed according to health record number and date. Two authors checked manually for errors in specialty assignment in respect to corresponding faculty and resident directory listings.

We reconciled missing data within the complication reporting section by assigning “no complication” in the absence of documentation to ensure even distribution of under-reporting bias. For this analysis, controls were designated as those with a negative test result and those who did not have a test sent to ascertain disease-specific variation in FPS and complication rates. Given limited testing capabilities early in the pandemic, patients were not universally tested and we assume this group was most similar to test-negative subjects due to low clinical suspicion.

### Statistical Analysis

We analyzed variables using JMP Pro, version 15 (SAS Institute, Cary, NC). Data were analyzed by intubation date and both persons under investigation and COVID-19 status. We used descriptive statistics with tests of association to analyze variables according to COVID-19 status. Variables within procedure notes were not mutually exclusive (i.e., multiple responses were possible and “no response” was also an option). We therefore chose analyze each specific variable across COVID-19 positive versus control status to compare incidences to avoid introducing bias. Tabulated data is therefore presented as column percentages and raw response rates. Categorical variables were summarized as number (%) and compared using a Pearson’s chi-square test or two-tailed Fisher’s exact test at a two-sided significance level of 0.05. The assumptions for the chi-square test were that the study groups were independent and that the sample size had cell count with >5 cases. Continuous variables are presented as medians and with a 95% confidence interval (CI).

A post-hoc sensitivity analysis of minimum effect size (MES) was conducted for the Fisher’s exact test assuming two independent samples (COVID-19 positive N = 123; COVID-19 negative N = 283) with an α = 0.05 and 1-β = 0.8. For the primary endpoint, FPS rate, we used a previous baseline FPS rate of 84% from unpublished institutional data and 89% from a recent report of emergency tracheal intubation in COVID-19 patients by Yao, Wang, and Jiang et al.^3^ This estimated baseline FPS rate is also consistent with a previous systematic review.^16^ We calculated a two-sided MES of ±2.6% for FPS rate. For our secondary endpoint, post-intubation complication rate, we selected hypoxia to calculate a MES for post-intubation complication rate. Yao and colleagues reported an hypoxia rate of 17.8% in COVID-19 tracheal intubations, which differs significantly from a rate of 6.4% (95% CI, 2.5–11.9) in a prior meta-analysis.^3^,^16^ We calculated a MES of ±9.4% for post-intubation complication rate. We additionally calculated an MES of ±15.1% for video laryngoscope as the initial laryngoscopy mode using a previous study, which reported an incidence rate of 52% in outside-of-OR tracheal intubations.^17^ All power calculations were performed using a statistical power analysis program G*Power 3.1.^18^ We did not report significance values for the subgroup analysis as this study as this study was not powered accordingly.

## RESULTS

**Figure 2:**
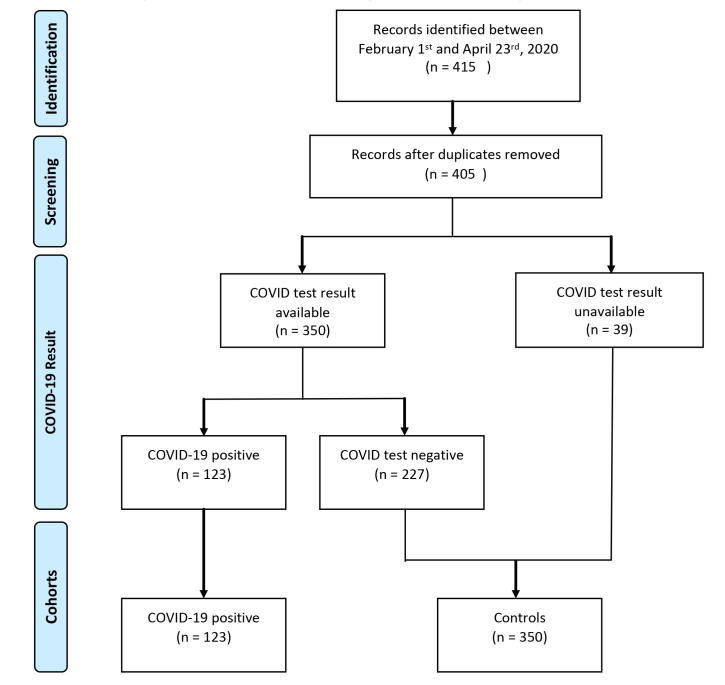
COVID-19-positive and control cohorts generated from procedure notes and COVID-19 test results collected from electronic health record intubation report between February 1–April 23, 2020.

**Figure 3:**
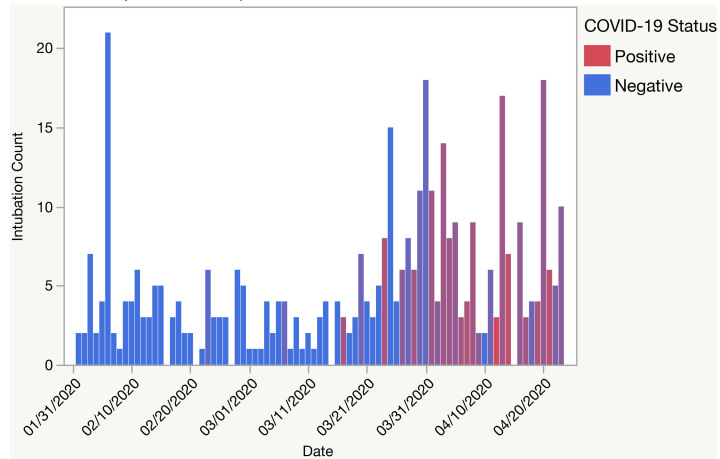
Count of non-operating room emergent intubations by date and COVID-19 test results at a single institution in Boston, MA, between February 1–April 18, 2020.

We identified 405 discrete procedure notes filed and 350 COVID test results between February 1–April 23, 2020 (Figure 2). On March 24, 2020, the total volume of non-OR emergent intubations increased from four to eight intubations per day and 3/8 (37.5%) would eventually test positive for COVID-19 (Figure 3). Most patients with a positive COVID-19 test result were intubated for respiratory failure (96.8% vs 71.7%) when compared to controls (Table 1). Control patients were more often intubated for airway compromise (25.2% vs 4.1%). A greater proportion of COVID-19 positive intubations were performed by CRNAs (37.4% and 11.7%, respectively), and fewer intubations of these cases were performed by both anesthesia and EM residents (10.5% and 15.5%, respectively) (Table 2). Anesthesiology performed more intubations in patients with a positive COVID-19 test result when compared with EM (Table 2).

The majority of intubation procedures in both groups were performed in designated ICU-level areas including OR and post-anesthesia care unit locations rather than the ED. (Table 3). A face mask was used more often (57.0% vs 34.6%) than nasal cannula for pre-oxygenation in COVID-19 cases compared to controls. Video laryngoscopy was used more frequently in patients who had a positive COVID-19 test result (91.1% vs 56.8%, ρ = <.0001).

An increased rate of oxygen desaturation was observed (20.3% vs 9.9%, ρ = 0.0061) in COVID-19 cases, but there were no significant differences in other peri-intubation adverse events (Table 4). However, COVID-19 intubations were performed less frequently in the ED compared to controls (38.2% vs 22.8%). Of note, no difference was found in the FPS rate between COVID-19 and control intubations (89.4% vs 89.0%, ρ = 1.0) as well as in the number of intubations requiring either 2 or >3 attempts.

We performed subgroup analysis to characterize trends in intubation performance (FPS), use of video laryngoscopy, and incidence of desaturation by provider types. First-pass success was obtained in 194/224 cases (86.7%) performed by residents compared to 168/182 (92.3%) performed by non-resident providers (anesthesiologist, EM attending, CRNA) (Table 5). Video laryngoscopy was used most often by CRNAs (98.7%) and least often by EPs (36.4%). A consistent trend of increased use of video laryngoscopy in COVID-19 positive cases was seen across all provider types except EPs (COVID-19 positive: 36.4% vs controls: 50.0%).

## DISCUSSION

For a period of 30 days from March 24–April 24, 2020, we observed a doubling in the number of emergent non-OR intubations performed daily at our institution with a majority attributed to COVID-19. We performed a retrospective analysis of all airway procedure notes documented at our hospital and have presented trends in clinical parameters that suggest adherence with recently published guidelines (eg, first attempt by expert laryngoscopist, preferential use of video laryngoscopy, RSI).^19^,^20^ For our primary endpoint, we did not find a significant difference in FPS rate between patients with a positive COVID-19 test result and those with either a negative test result. Of note, we found an average FPS rate of 89%, which is higher than our institutional baseline of 84%. This is comparable to the FPS rate (89%) reported in a case series in Wuhan, China. This may be explained by a number of variables. Intubating clinicians may have been more aware of the possibility of a difficult airway with rapidly progressing hypoxia due to institutional meetings or widely distributed literature and likely made changes to their practice with the primary goal of maximizing FPS. An example is that most clinicians began routinely using video laryngoscopy in suspected COVID-19 cases. Although all emergent intubations hospital-wide were subject to similar isolation procedures, the selection of technique and induction medications were subject to clinician preference.

The role of trainees during the pandemic was the subject of much discussion in our institution as it was internationally. Striking an ethical balance between potential exposure and the need for supervised experiential learning continues to be debated. We observed a decreased proportion of COVID-19 intubations performed by trainees and an increase in those performed by non-trainee providers, particularly CRNAs. This can be attributed to the reconfiguration of the anesthesia emergency airway response team prior to March 24. The ED intubation team remained as an attending and senior airway resident, with attending discretion on laryngoscopist. Of note, both EM and anesthesia attending success rates were the lowest in their fields. This is alarming at first glance, but we hypothesize this was largely due to the attending preferentially performing the more difficult intubations, which included cases in which the patients were profoundly hypoxemic and/or morbidly obese.

A higher percentage of intubations involving COVID-19 patients were performed by anesthesia when compared to EM. Since EM, with rare exception, performs all intubations in the ED, this statistic suggests that a higher percentage of COVID-19 patients deteriorated once admitted, and subsequently required intubation. This is congruent with our internal unpublished data that indicates 53% of our COVID-19 ICU admissions originated as transfers from the wards (47% occurring directly from the ED). Although some intubations performed by anesthesia represent repeat intubations such as a tube changes or re-intubation after unplanned/failed extubation, it is unknown whether the incidence of repeat intubations could have been higher in the COVID-19 group. These repeat intubations were not excluded in this study and may present a different likelihood of FPS.

We found increased utilization of video laryngoscopy in COVID-19 cases that is consistent with recent expert recommendations.^21^,^22^ It is important to note that these guidelines were motivated by an effort to reduce provider infections by increasing distance between patient and operator rather than intubation performance. There is mixed evidence that video laryngoscopy results in decreased intubation attempts or reduced incidence of peri-intubation hypoxia.^23^ We did not perform a stratified analysis of FPS rate by use of video laryngoscopy due to our limited sample size.

The use of several preoxygenation modalities including high-flow nasal cannula, non-invasive positive pressure ventilation (NIPPV) and bag valve mask (BVM) were linked to aerosolization and increased risk of nosocomial infection during the 2003 SARS epidemic.^24^ Society guidelines have recommended against positive pressure ventilation for preoxygenation unless clinically indicated.^21^ We did not observe a statistically significant decrease in the use of manual positive pressure ventilation for preoxygenation (ie, BVM or NIPPV). A new method of preoxygenation for patients with suspected COVID-19 infection that was not captured in the structured text fields of our airway note is the specific use of a bag-mask with positive end-expiratory pressure valve and viral filter to administer 100% oxygen via continuous positive airway pressure (without “bagging” or administering ventilation). This technique has been described by Weingart.^23^ Review of the free-text fields of airway notes (as well as the authors’ experience) indicate that this preoxygenation method was widely employed by both anesthesia and emergency clinicians. We additionally did find a measurable decrease in the overall use of NIPPV of 4.6%, which is considerably lower than 70.8% reported in Wuhan, China.

## LIMITATIONS

We attempted to compare unmatched cases by selecting a temporal cohort during which a standardized intubation protocol had been implemented. However, we acknowledge the limitation of retrospective analysis as our methodology.^25^ Selected agents for both sedation and paralysis were also not standardized and were subject to provider preference. This study does not confirm whether patients with COVID-19 present specific physiologic barriers to ETI or alternative intubation methods are more or less successful. Our study had a limited sample size and was not adequately powered to detect a difference in FPS less than 12.6%. Another limitation was related to reporting of COVID-19 test results within our electronic health record and the lag time for results. We cannot exclude whether these patients were intubated according to institution COVID-19 protocols (negative pressure room, video laryngoscopy, expert laryngoscopist).

We acknowledge that this study did not involve chart review and that no additional quality analysis was performed to ensure complete capture of emergent intubations or complications during the study period. The authors served as data abstractors in our study and were not blinded to the study hypothesis. A potential under-reporting bias was introduced when missing complication data was assigned to the null. However, we anticipate that this would be evenly distributed among both COVID-positive and negative cases. Additionally, the authors were not blinded during data appraisal. Our study was secondarily limited in that the definition of intubation attempt was not determined a priori, and variation among providers is likely present. For our post-intubation complication rates, reporting bias is possible with complication documentation, as under-reporting in health records is a known phenomenon. The assumption that this bias is equally distributed between COVID-positive and controls was not determined.^26^ A selection bias is likely also present as trainees may be given the opportunity to perform laryngoscopy more often in patients with less difficult airways. Additionally, tube exchanges were not excluded from this analysis and may have biased FPS rates.

## CONCLUSION

We observed a significant increase in the total volume of emergent intubations performed at a single center in Boston, Massachusetts, during the 30-day period March 24–April 24^th^, 2020. We found that first-pass success and complications other than oxygen desaturation were not significantly different between COVID-19 positive cases and controls. Future prospective trials should investigate factors surrounding emergency airway management including team composition and video laryngoscopy on intubation performance.

## Supplementary Information



## Figures and Tables

**Table 1:** Patient characteristics associated with COVID-19 compared with controls. Data presented as column percentages and raw number of annotated responses. An asterisk indicates where α < 0.05.

	COVID-19 positive (n = 123)	Controls (n = 283)	P-value
Indication			
% Respiratory failure, (n)	96.8%, (119)	71.7%, (203)	<.0001*
% Airway compromise, (n)	4.1%, (5)	25.4%, (72)	<.0001*
% Cardiac arrest, (n)	0%, (0)	5.0%, (14)	0.0073*
Cormack–Lehane grading			
% Grade 1, (n)	82.1%, (101)	73.5%, (208)	0.0758
% Grade 2, (n)	12.2%, (15)	18.0 %, (51)	0.1872
% Grade 3, (n)	4.9%, (6)	5.7 %, (16)	1.0
% Grade 4, (n)	0%, (0)	1.4%, (4)	0.3195

*COVID-19*, coronavirus disease 2019.

**Table 2:** Provider characteristics associated with COVID-19 compared with controls. An asterisk indicates where α < 0.05.

	COVID-19 positive (n = 123)	Controls (n = 283)	P-value
Specialty			
% Anesthesiology, (n)	75.6%, (93)	61.8%, (175)	0.0086*
% Emergency Medicine, (n)	22.8%, (28)	38.1%, (108)	0.002*
% Pulmonary Critical Care, (n)	1.6%, (2)	0%, (0)	-
Provider type			
% Anesthesiology resident, (n)	10.5%, (13)	33.9%, (96)	<.0001*
% EM resident	15.5%, (19)	33.9% (96)	<.0001*
% Anesthesiologist, (n)	27.6%, (34)	16.3%, (46)	0.0099*
% CRNA, (n)	37.4%, (46)	11.7%, (33)	<.0001*
% Emergency physician, (n)	7.3%, (9)	4.2%, (12)	0.0656
% Non-emergency physician, (n)	1.6%, (2)	0%, (0)	

*COVID-19*, coronavirus disease 2019; *EM*, emergency medicine; *CRNA*, certified registered nurse anesthetist

**Table 3:** Location, selection of pharmacologic agents intubation technique associated with COVID-19 compared with controls. An asterisk indicates where α < 0.05.

	COVID-19 positive (n = 123)	Controls (n = 283)	P-value
Location of intubation			
% ICU (n)	69.1%, (85)	60.4%, (171)	0.1171
% ED (n)	22.8%, (28)	38.2%, (108)	0.0028*
Medication			
Induction agent			
% Propofol, (n)	74.5%, (91)	47.4%, (134)	<.0001*
% Fentanyl, (n)	0%, (0)	0.71%, (2)	-
% Etomidate, (n)	22.0%, (27)	34.6%, (98)	0.0138*
% Ketamine, (n)	3.3%, (4)	8.8%, (25)	0.0574
% Midazolam, (n)	0%, (0)	0%, (0)	-
% No induction agent, (n)	0.8%, (1)	9.5%, (27)	0.0005
Paralytic			
% Succinylcholine, (n)	1.6%, (2)	46.4%, (123)	<.0001*
% Rocuronium, (n)	98.4%, (121)	52.4%, (139)	<.0001*
% Vecuronium, (n)	0%, (0)	1.1%, (3)	-
Technique			
% Rapid Sequence Intubation	99.2%, (122)	84.5%, (239)	<.0002*
Preoxygenation method			
% BVM, (n)	29.2%, (36)	38.2%, (108)	0.0913
% Facemask, (n)	57.0%, (70)	34.6%, (98)	<.0001*
% Nasal cannula (n)	12.2%, (15)	22.6%, (64)	0.014*
% Non-invasive positive pressure ventilation, (n)	1.6%, (2)	5.7%, (16)	0.1118
% Apneic oxygenation, (n)	8.1%, (10)	11.7%, (33)	0.3802
Laryngoscopy and tube confirmation			
% Video laryngoscopy, (n)	91.1%, (75)	56.8%, (130)	<.0001*
% Use end-tidal CO_2_	22.2%, (90)	46.8%, (190)	0.2447

*COVID-19*, coronavirus disease 2019; *ICU*, intensive care unit; *ED*, emergency department; *BVM*, bag valve mask; *CO_2_*, carbon dioxide.

**Table 4:** First-pass success, multiple attempts, and incidence of complications associated with COVID-19 compared with controls. An asterisk indicates where α < 0.05.

	COVID-19 positive (n = 123)	Controls (n = 283)	P-value
Number of Attempts			
% First-pass success, (n)	89.4%, (110)	89.0%, (252)	1.0
% 2 attempts	8.1%, (10)	10.3%, (29)	0.5853
% >3 attempts	2.4%, (3)	0.7%, (2)	0.5853
Adverse events			
% Dysrhythmia, (n)	2.4%, (3)	0.4%, (1)	0.0849
% Hypotension, (n)	9.0%, (11)	5.0%, (14)	0.1755
% Cardiac arrest, (n)	1.6%, (2)	1.8%, (5)	1.0
% Aspiration, (n)	0%, (0)	0.7%, (2)	1.0
% Desaturation, (n)	20.3%, (25)	9.9%, (28)	0.0061*
% Esophageal intubation, (n)	0%, (0)	0%, (0)	-
% Laryngospasm, (n)	0%, (0)	0%, (0)	-
% Bronchial intubation, (n)	0%, (0)	0%, (0)	-
% Airway trauma, (n)	0%, (0)	0%, (0)	-
% Dental trauma, (n)	0%, (0)	0%, (0)	-
% Medication error, (n)	0%, (0)	0%, (0)	-
% Equipment failure, (n)	0.8%, (1)	0.7%, (2)	1.0
% Any complication, (n)	8.1%, (10)	13.4%, (38)	0.1365

*COVID-19*, coronavirus disease 2019.

**Table 5:** Subgroup analysis of first-pass success rate and video laryngoscopy use by provider type and COVID-19 status.

	Anesthesiologist (n=80)		EM physician (n=23)		Anesthesiology resident (n=109)	
			
	COVID-19 positive	Controls	COVID-19 positive	Controls	COVID-19 positive	Controls
% First pass success, (n)	94.1% (32)	91.3% (42)	63.6% (7)	91.6% (11)	76.9% (10)	90.9% (100)
% Video laryngoscopy, (n)	94.1% (32)	50.0% (23)	36.4% (4)	50.0% (6)	100% (13)	88.2% (97)
% Desaturation, (n)	20.6% (7)	6.5% (3)	27.3% (3)	16.7% (2)	38.5% (5)	8.3% (8)

*COVID-19*, coronavirus disease 2019; *EM*, emergency medicine.
