# Supplementary material for: Emergency Tracheal Intubation in Patients with COVID-19: A Single-center, Retrospective Cohort Study
Source: West J Emerg Med. 2021 May 17;22(3):678–86. doi: 10.5811/westjem.2020.2.49665 (PMC8203023; doi:10.5811/westjem.2020.2.49665)
Supplement: Supplementary file 1 [file wjem-22-678-s001.docx]

**Appendix A**

Recommendations for Airway Management during the COVID-19 Pandemic

Purpose

COVID-19 may be asymptomatic and contagious. There is a limited supply of appropriate Personal Protective Equipment dictating that we differentiate between SUSPECTED OR CONFIRMED COVID-19 patients and NON-COVID SUPSPECTED patients.

This guideline describes how to manage the airway of all patients admitted to the hospital and requiring endotracheal intubation during the COVID-19 pandemic. Additionally, it details airway management in non-intubated and intubated Suspected or Confirmed COVID-19 patients requiring an urgent procedure. It also describes how to manage patients coming for urgent procedures that have been screened and are determined to be Non-COVID Suspected patients.

**Team Members**

- Primary Laryngoscopist
- Supporting airway provider
- Respiratory Therapist
- Bedside Nurse

**Personal Protective Equipment**: Enhanced precautions (contact, airborne, droplet)

- Inner gloves: Disposable medical gloves
- Outer gloves: Disposable medical gloves with extended cuffs or sterile surgical gloves
- Gown: Impermeable with integrated thumb hooks
- N95 or PAPR (Powered Air Purifying Respirator)*
- Hood
- Face shield
- Footwear covers (optional)

*PAPRs are available for those health care workers that have not been fit-tested or cannot wear the N95 respirator. To obtain a PAPR, call Transport at 4-5835. The Transport Center will ask for caller’s name and the location where the PAPR is to be delivered.

Follow the Donning and Doffing for Airway Management Guidelines with observer-ensured compliance.

**Airway Management Equipment**

Use the dedicated COVID-19 Airway Management Kit (adult or pediatric). These COVID-19 Airway Management Kits will be in the Anesthesia Workroom in labeled orange tackle boxes. The COVID Glidescopes must be plugged in when not in use to ensure full battery charge.

Adult Kit contents:

- Outer gloves (Size 7 and 8) with extended wrist cuff
- Gown: Impermeable with integrated thumb hooks
- N-95 Respirator size regular only
- Hood
- Face shield
- Footwear covers (optional)
- 1 DIRTY red biohazard plastic bag
- 1 CLEAN red biohazard plastic bag
- GlideScope monitor
- Size 3 blade
- Size 4 blade
- One re-useable adult stylet
- One disposable pediatric stylet
- One re-usable adult camera/cable
- One HEPA filter
- Bleach wipes

Pediatric Kit contents:

- Outer gloves (Size 7 and 8) with extended wrist cuff
- Gown: Impermeable with integrated thumb hooks
- N-95 Respirator sizes small and regular
- Hood
- Face shield
- Footwear covers (optional)
- Red Biohazard sealable plastic bag
- GlideScope monitor
- Size 1 blade
- Size 2 blade
- One disposable pediatric stylet
- One re-usable pediatric camera/cable
- One HEPA filter
- Bleach wipes

Anesthesia Code Box: DO NOT TAKE CODE BOX INTO PATIENT’S ROOM. Contains medications and backup airway supplies for use as needed.

Emergency Department (ED) physicians will use ED equipment which is kept in the trauma rooms along with “To go” kits/pouches.

Anesthesia Medication Kit: etomidate, propofol, succinylcholine, rocuronium, phenylephrine, ephedrine, and sugammadex.

**Suspected or Confirmed COVID-19 Patients – Airway Management**

In patients that have developed respiratory failure requiring oxygenation and ventilation support, early tracheal intubation is recommended. Noninvasive positive pressure ventilation (NIPPV) or high flow nasal oxygen (HFNO) is NOT recommended and should be discontinued before attempting intubation to minimize exposure. NIPPV and HFNO both risk aerosolization of upper airway droplets that contain virus.

There are no “STAT” intubations in Suspected or Confirmed COVID-19 patients; donning and doffing PPE and preparing supplies to be brought into the patient’s room takes time. During nights and weekends, the in-house anesthesiologist must consider requesting anesthesia backup personnel (in-house or from the call team) to assist with the procedure or other anesthesia-related activities.

All intubations and extubations, to the extent possible, should be performed in a negative pressure room. Intubations should be performed by the most experienced available clinician.

Complete the routine preparation checklist including:

- Suction
- Ventilator setup
- IV access with ability to administer medications easily
- Standard monitors (BP, Oximeter, EKG)
- Draw up medications

The primary team should place ventilator and sedation orders prior to intubation to minimize the risk of bucking, coughing, and agitation after intubation. This lowers the risk of ventilator disconnection and accidental extubation, reducing the chance of aerosol contamination.

Bring only the items you intend to use into the room. All other items should remain outside of the room with the support team.

Example of items to be brought into the room:

- Medications you intend to use, drawn up outside the room with blunt needles attached
- Multiple 10 ml saline flushes with blunt needles attached
- GlideScope with appropriate blade and camera
- GlideScope stylet
- Endotracheal Tube
- 1 Oral Airway
- 1 DIRTY red biohazard bag to place used GlideScope, stylet, and reusable face shield

Items to be left outside the room with the support team:

- Anesthesia Code Box
- COVID airway management tackle box
- Medications you do not intend to use
- 1 CLEAN red biohazard bag into which the DIRTY biohazard bag will be placed

Use awake intubation ONLY when absolutely necessary. NOTE: Atomized local anesthetic will aerosolize the virus. Avoid nebulized medication administration whenever possible.

ED intubations should follow the above steps where pertinent with the following ED specific caveats:

- While expert opinion advises early intubation in critically ill COVID-19 cases, timing and necessity are matters of judgement. The ED should emergently consult pulmonary critical care regarding the need, urgency and timing of intubation and consensus reached. If the intubation is needed in the ED, then anesthesia should be consulted and the ED and anesthesia attending confer regarding the airway management including who will be the “Primary Laryngoscopist” and who the “Supporting Airway Provider”. In most cases, the Primary Laryngoscopist will be the anesthesia attending. In the rare case of a truly emergent intubation precluding the above processes, the most experienced Laryngoscopist immediately available (from ED) will manage the airway with supporting ED airway provider and adhering to PPE.
- ED intubations, should ideally take place in a negative pressure room (A3, B10 or B11). If no negative pressure room is available, then intubate in an available trauma room.
- Pharmacists will hand off medications outside to inside rooms (non-trauma rooms).

**Recommended Steps:**

Preoxygenate with 100% oxygen at 15 liters/min for 3-5 minutes of tidal breathing via Non-Rebreather Mask or Face mask attached to HEPA filter and AMBU bag (hold mask firmly on face).

Rapid Sequence Induction (RSI) is recommended for securing the airway to mitigate viral spread. Consider using rocuronium (1.2 mg/kg) to ensure paralysis after intubation and allow the ICU team to establish sedation and other care without rushing. Allow adequate time for NMBA onset, and DO NOT attempt to manipulate the airway until certain of neuromuscular block.

Avoid bag mask ventilation. If bag mask ventilation is necessary, consider placing an oral airway and use a two-handed technique to ensure maximum seal. Use low volume and higher frequency ventilation via bag-mask. Place a HEPA filter between the mask and bag.

Use a GlideScope to maximize first attempt success and allow for increased distance between the laryngoscopist and the patient’s mouth. After GlideScope intubation, consider attaching the endotracheal tube directly to the ventilator circuit and the attached HEPA filter. If you have a clear view of ETT passing through cords, rather than colorimetric CO2 detection, rely on the ventilator’s capnography if available. Do not use the AMBU bag for alveolar recruitment unless necessary. This strategy avoids multiple disconnects. In the Emergency Department, continuous wave capnography is the preferred method to confirm adequate ventilation using colorimetric CO2 detection only as back-up.

All disposable airway equipment should be discarded in a designated container prior to leaving the patient’s room.

Non-disposable equipment including GlideScope monitor, GlideScope camera, stylet, and reusable face shields will be placed in the DIRTY red biohazard bag in the patient’s room. Eventually, the DIRTY biohazard red bag will be placed into a CLEAN biohazard bag and brought to the dirty utility room in the OR for disinfection by the Anesthesia Technicians. After initial disinfection, the Anesthesia Technicians will send the GlideScope stylet to CPD for final processing and reassemble to COVID Airway Management Kit with clean supplies.

Remove your PPE by following the Donning and Doffing for Airway Management Guidelines.

**Additional Considerations:**

In patients that have features suggesting difficult airway, consider having the supporting airway provider fully donned in PPE outside the room for immediate assistance. The supporting airway provider should enter the room if summoned by the primary laryngoscopist.

Limit ventilator disconnects. If you need to disconnect the ventilator for any reason, do so proximal to the HEPA filter.

**Non-COVID Suspected Patients – Airway Management**

Endotracheal intubation is considered a procedure that is high risk for virus transmission. All Non-COVID Suspected patients requiring endotracheal intubation throughout the hospital should be intubated with the same precautions and PPE as Suspected or Confirmed COVID-19 patients.

**Suspected or Confirmed COVID-19 Patients Requiring an Urgent Operation**

Operating rooms 3 and 4 are designated COVID-19 operating rooms. The amount of surgical and anesthesia supplies within these rooms is reduced to limit contamination.

If already intubated, Suspected or Confirmed COVID-19 patients that require a procedure should be transported from the intensive care unit (ICU) directly to IPP 3, 4, or the appropriate IPP location by personnel donning PPE. Use an Ambu bag with a HEPA filter between the Ambu bag and endotracheal tube for transport. After the patient is moved to the operating room table, the patient’s hospital bed should remain in the operating room to limit the number of times the OR doors are opened.

If not intubated, the patient will be induced and intubated in IPP 20 (negative pressure room) following the above recommendations. The patient will then be transferred to OR 3, 4, or the appropriate IPP location by the team wearing PPE and with a HEPA filter between the Ambu bag and the endotracheal tube. After the procedure, if the patient remains intubated, they will be transported to their final destination with an Ambu bag and HEPA filter. If extubation is planned, the patient will be transported to PACU room 27 (negative pressure room) for extubation and recovery following the same transport protocol.

**Non-COVID-19 Suspected Patients Requiring Procedures – Airway Management**

Non-COVID-19 Suspected patients undergoing urgent surgical or endoscopic procedures will be screened for COVID-19 symptoms and exposures. The following PPE should continue to be used for procedures requiring intubation:

- Level 3 Cardinal Health Surgical Mask with Anti-Fog Foam Strip and Wraparound Eye Shield (This mask provides enhanced protection as compared to the other surgical masks available)
- Yellow or blue gown
- Nonsterile double gloves
